# A rare case of synchronous breast cancer metastasis to colonic and gastric mucosa—case report and review of literature

**DOI:** 10.1093/omcr/omaf181

**Published:** 2025-09-28

**Authors:** Zoha Asghar, Zubaid Moazzam Sheikh, Lubna Saleem

**Affiliations:** Department of Medicine and Surgery, Aga Khan University Hospital, Karachi 74800, Pakistan; Department of Medicine and Surgery, Aga Khan University Hospital, Karachi 74800, Pakistan; Department of Medical Oncology, Cancer Foundation Hospital, Karachi 74800, Pakistan

**Keywords:** breast carcinoma, metastasis, gastrointestinal symptoms, infiltrative lobular carcinoma

## Abstract

Distant metastasis from primary breast cancer typically affects the brain, liver, lungs, and bones. Less than 1% of patients exhibit metastasis to the stomach or colon, mimicking primary gastrointestinal tumors upon initial presentation. Here, we present the case of a middle-aged female who initially presented with synchronous breast cancer and metastasis to the gastrointestinal tract. A middle-aged female presented to our center with GI symptoms. Prior upper GI endoscopy showed diffuse infiltration of atypical tumor cells resembling poorly cohesive adenocarcinoma of the stomach. Immunohistochemical stains were positive for markers indicative of breast origin, and negative for CDX2 and TTF1, ruling out gastrointestinal and lung origins, respectively. Breast ultrasound confirmed architectural disruption, and suspicious bilateral axillary lymph nodes. Biopsy of the axillary lymph nodes confirmed the presence of breast cancer cells. She was treated with Letrozole and Ribociclib showing complete resolution on subsequent CT scans. The patient continues this regimen and is now in clinical and radiological remission for the past four years.

## Introduction

Distant metastasis from primary breast cancer is a cause of significant mortality and most common sites involved are brain, liver, lungs and bone. However, in less than 1% patients we see an unusual presentation of metastasis to stomach and colonic mucosa mimicking a primary gastric or colonic tumor which can be difficult to differentiate on clinical presentation as patients often present with non-specific abdominal symptoms such as diarrhea, vomiting, obstruction, abdominal pain and distention [1]. In general, in most cases of breast cancer distant metastasis occurs after years of a primary diagnosis but around 5–8% of patients can present with de novo metastatic disease [2]. The most common location of breast cancer metastasis in GIT is the stomach, which occurs commonly in invasive lobular carcinoma (ILC) histologic subtype [3]. Since many patients present with vague clinical symptoms and variable pathology on endoscopy, diagnosing GI metastasis is difficult, necessitating tissue samples for histopathologic confirmation [4]. We report a rare case of Infiltrative Lobular Carcinoma of the breast presenting with metastasis to the stomach, duodenum and colonic mucosa where the chief complaints of the patient were that of the gastrointestinal tract without a prior diagnosis of breast cancer.

## Case report

A 45-year-old female with a history of Diabetes Mellitus for 5 years and Hypertension for 11 years, presented to our center with complaints of abdominal distension, nausea, vomiting and altered bowel habits for 2–3 months. The patient reported no history of performing self-breast examination and denied any known family history of malignancy. Her BRCA mutation status remains undetermined. On a complete physical exam, both breasts were found to be firm without any palpable lump or axillary lymph node, however supraclavicular and inguinal lymph nodes were palpable, abdomen was distended and multiple lumps in the upper abdomen were palpable. To provide symptomatic relief, multiple therapeutic ascitic taps were done over the last 2–3 months due to large recuring ascites. Ascitic fluid testing showed exudative fluid with a negative cytology. She also reported one episode of per-rectal bleeding in the past.

**Figure 1:**
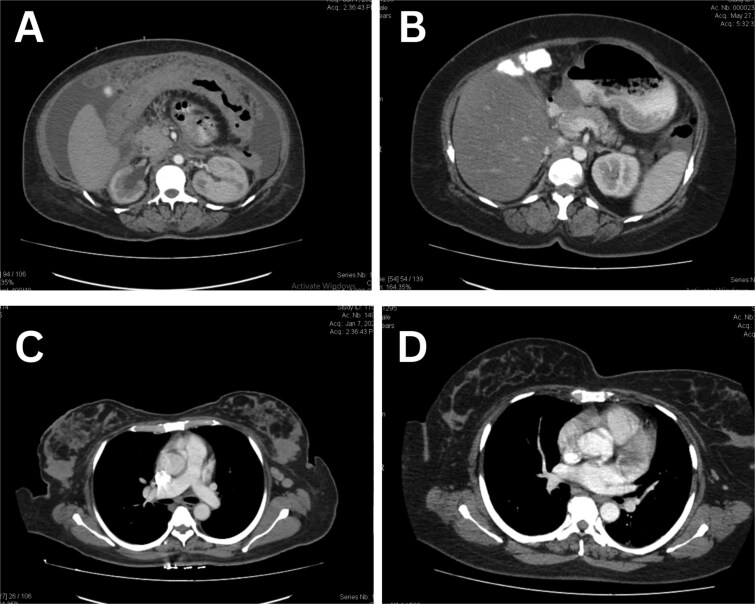
Breast tissue and gastric mucosa before and after treatment. (A) Omental caking, ascites, gastric mucosal thickening and carcinomatosis. (B) Resolution of carcinomatosis in the gastric mucosa. (C) Breast tissue with carcinoma. (D) Resolution of carcinoma in breast tissue.

**Figure 2:**
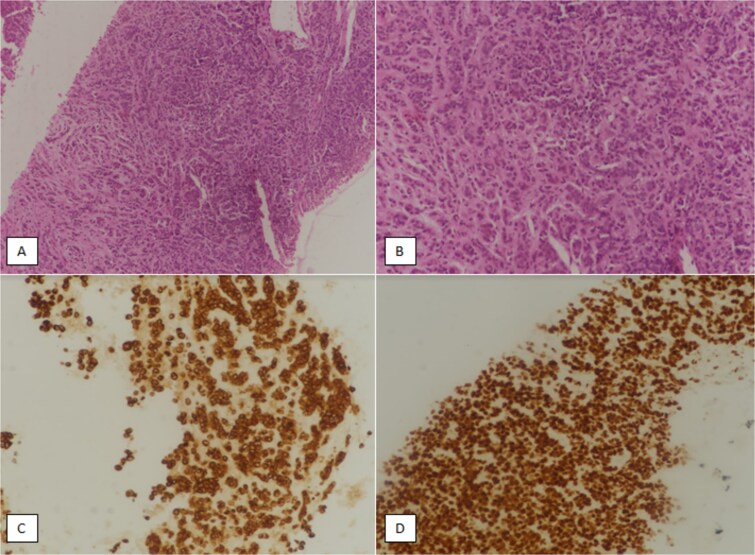
(A) Hematoxylin and eosin (H & E)-stained section at 100× magnification showing fibro-collagenous tissue diffusely infiltrated by atypical cells. (B) H & E-stained section at higher magnification (200×) showing tumor cells present in nests, clusters, and as individual cells. Tumor cells are medium sized with atypical hyperchromatic nuclei. (C) Immunohistochemical (IHC) staining for cytokeratin 7 (CK7) at 200× magnification showing positive staining with CK7 marker. (D) IHC staining for GATA binding protein 3 (GATA3) at 200× magnification showing diffuse positivity with GATA3.

The patient did not have a prior breast ultrasound or mammogram although she brought a CT scan of abdomen and pelvis with her which reported focal thickening of pylorus, circumferential wall thickening of the entire colon, and omental thickening and caking were present with an anteroposterior diameter of 27.7 mm. Furthermore, multiple paraaortic, inguinal and pelvic lymph node involvement was seen on CT scan (Fig. 1). Upper and lower GI endoscopy showed gastric and bowel wall oedema, multiple strictures in colon. Endoscopic samples from stomach, duodenum and colon were taken for biopsy which revealed gastric, small bowel, and large bowel mucosa with effaced architecture. The lamina propria were diffusely infiltrated with atypical tumor cells. These tumor cells had an appearance mimicking poorly cohesive adenocarcinoma of the stomach. Immunohistochemical (IHC) stains were performed on the biopsy material. The tumor cells stained positive with cytokeratin (CK) 7, and GATA binding protein 3 (GATA3) (a maker of breast origin), while negative with CDX2 (marker of gastrointestinal origin) and TTF1 (marker of lung origin). Based on IHC profile, metastatic carcinoma from breast was favored (Fig. 2). There were also signet ring cells present in submucosa so poorly differentiated adenocarcinoma with signet ring cells morphology likely metastasis from breast was the final pathology.

**Figure 3:**
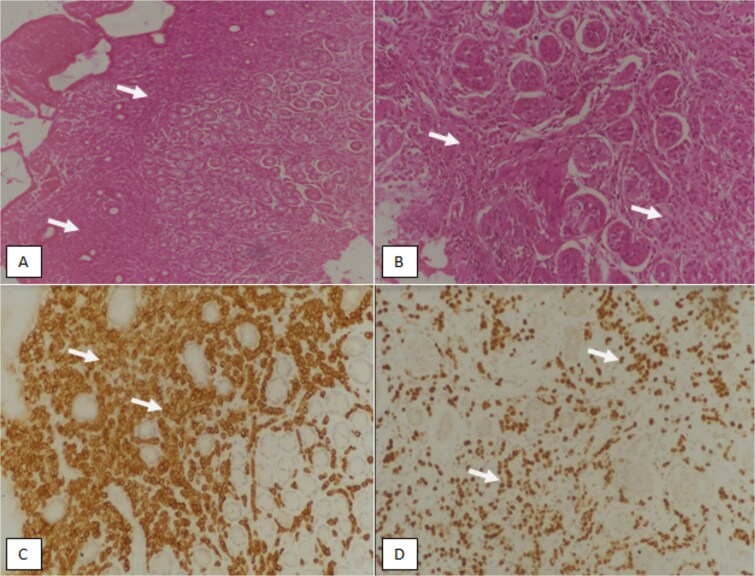
(A) Hematoxylin and eosin (H & E)-stained section at 100X magnification showing effaced gastric mucosa diffusely infiltrated by malignant cells in the lamina propria (arrows). (B) H & E-stained section at higher magnification (200×) showing tumor cells in between the gastric glands (arrows). (C) Immunohistochemical (IHC) staining for cytokeratin 7 (CK7) at 200× magnification highlighting tumor cells in the lamina propria (arrows). Gastric glands are not stained by CK7 stains. (D) IHC staining for GATA binding protein 3 (GATA3) at 200X magnification depicting tumor cells in the lamina propria (arrows).

Following the results of IHC and histopathology a breast ultrasound was done which further revealed diffuse inflammatory changes, architectural disruption, edema and skin changes in both breasts and suspicious bilateral axillary lymph nodes. Therefore, an ultrasound guided true cut biopsy of axillary lymph nodes was performed. Histology showed a metastatic, poorly differentiated carcinoma with signet ring morphology was that consisted of atypical cells with abundant eosinophilic cytoplasm and hyperchromatic nuclei (Fig. 3). IHC was positive for CKAE1/AE3, CK7, ER, PR and Her2neu 1 +, congruent to the IHC findings of gastric mucosa biopsy.

There was no visceral metastasis, but the bone scan demonstrated areas of uptake in thoracic vertebra. The patient was started on chemotherapy with single-agent docetaxel for 18 weeks but due to poor tolerance was switched to a hormonal combination of Letrozole (selective aromatase inhibitor) and Ribociclib (CDK 4/6 inhibitors) in July 2020. This hormonal combination worked excellent in her case and subsequent CT scans showed complete resolution at all sites of involvement. The patient has been on this combination for last 4 years and in clinical and radiological remission. Below are the images of her initial CT scan showing diffuse both breast thickening, edema and enlarged axillary nodes, omental thickening and bowel wall edema which on subsequent CT scans is resolved (Fig. 3).

## Discussion

Metastasis from breast cancer to a distant site could be either to a usual site such as lung, liver or bone or an unusual site such as GIT [5]. Breast Cancer patients can also, in very rare cases, present initially with metastasis to the skin [6].

It can present as denovo metastatic disease or as a recurrence years after the treatment, the former being more common. There exist only a few reports to the best of our knowledge regarding metastasis to the GIT in breast cancer or GI symptoms preceding breast cancer diagnosis. The number of reports highlighting peritoneal carcinomatosis, gastric and/or colonic involvement is also limited. Our study is one of the few to report gastric, colonic and duodenal involvement in conjunction with peritoneal carcinomatosis. In addition, in our case the patient did not have an established diagnosis of breast cancer and presenting complaints vaguely suggested a gastric carcinoma with peritoneal carcinomatosis.

Infiltrating lobular Carcinomas (ILCs) account for about 10–15% of all breast cancers and have a different metastatic pattern as compared to intraductal carcinomas which can be attributed to the loss of E-cadherin expression on tumor cell membrane, promoting invasion and preventing cell to cell attachment [7]. According to the Mayo Clinic data, among 12 000 cases of metastatic breast cancer, 73 cases had GI metastasis. Invasive Lobular Carcinoma is shown to have frequent distant metastases as compared to invasive ductal carcinoma which is in-line with our findings [8, 9].

The stomach presented as the most common site of distant metastasis of GIT for breast cancer in our literature review however incidence remains significantly low, 0.2–0.7% [10, 11]. A retrospective cohort study spanning over 13 years, from 1995 to 2008 reported 8 cases of gastric metastasis from a primary breast tumor all showing lobular histology which is also the case in our presentation [11].

In our case report the pathology of axillary node and GIT mucosa both were that of poorly differentiated carcinoma with signet ring cell type, which is usually a GIT mucosa pathology and rarely reported in breast cancer. Bakker et al. reported a similar case in 2016 where a patient presented with isolated diarrhea caused by diffuse metastatic lobular cancer and on colonoscopy showed a normal colonic mucosa, but microscopic examination revealed infiltration of neoplastic cells in the lamina propria and biopsy showing cells resembling those of lobular carcinoma with signet ring histology [8]. This should alert physicians about the indication of biopsy in the presence of relatively normal endoscopic findings in an endoscopy and/or colonoscopy. It is rare to find a gastric metastasis with a histology resembling a primary gastric tumor such as signet ring morphology which is present in our case. In some cases, gastric metastasis can also be the only site of metastasis like in our case. Tetsuji Kudo et al. reported a case of solitary minute metastasis from breast cancer that can mimic primary gastric cancer, their histology also showed signet ring cell carcinoma. Subsequent tumor markers were positive for GCDFP15 which is specific for breast cancer [12]. Tumor markers can also aid in differentiating among breast, colon, and gastric cancers due to their varying expression, particularly CK7 and CK20. Most breast cancers are positive for CK7 and negative for CK20 whereas most GI cancers are negative for CK7 and positive for CK20 [13].

## Conclusion

Although a rare occurrence, metastatic disease should remain on the differential for patients with breast cancer presenting with signs and symptoms of common GI disorders. This warrants a screening colonoscopy and/or endoscopy of patients treated for primary breast cancer to rule out metastasis to the stomach and colonic mucosa, especially in the presence of abdominal symptoms.

## Data Availability

Data available per request.
